# A Patient’s Journey With Rare Biphenotypic Hepatocellular Carcinoma and Cholangiocarcinoma

**DOI:** 10.7759/cureus.9838

**Published:** 2020-08-18

**Authors:** Danny B Gersowsky, Kamran Mohiuddin, Cynthia Deasey, Joanne Cipollini, Madiha Gilani

**Affiliations:** 1 Medicine, Albert Einstein Healthcare Network, Philadelphia, USA; 2 Emergency/Internal Medicine, Albert Einstein Healthcare Network, Philadelphia, USA; 3 Oncology, Albert Einstein Healthcare Network, Philadelphia, USA

**Keywords:** hcc, cholangiocarcinoma, hepatocellular carcinoma, biphenotypic

## Abstract

Combined hepatocellular-cholangiocarcinoma (cHCC-CC) is a rare neoplastic primary liver cancer that is also known as mixed HCC-CC since it portrays characteristics of both hepatocellular carcinoma (HCC) and cholangiocarcinoma (CC). It constitutes less than 5% of primary liver cancers, hence, the literature lacks guidance on the management of these patients. A handful of case series has been published on clinical features and surgical outcomes. There is next-to-no mention of how to treat these patients. However, surgery has proven the most definitive treatment with varied responses to systemic therapies. We present a case of cHCC-CC in a patient who has undergone multiple treatment modalities, including surgical resection, chemotherapy, immunotherapy, and targeted therapy.

## Introduction

While little is known about these tumors, it is hypothesized that combined hepatocellular-cholangiocarcinoma (cHCC-CC) tumors arise from hepatic progenitor cells, which can lead to biphenotypic features. While the clinical characteristics of cHCC-CC are similar to those of hepatocellular carcinoma (HCC), the survival rates are similar to cholangiocarcinoma (CC) [1]. When viewed with a deeper lens, cHCC-CC can be further categorized into three types. Type A is when HCC and CC exist separately and is commonly called “double cancer.” Type B represents cases in which HCC and CC are located nearby but still independently. Type C is when components of HCC and CC intermingle within the same tumor; this is commonly called the mixed type and is considered the “true” biphenotypic cHCC-CC. Thus, biphenotypic cHCC-CC is commonly referred to as just cHCC-CC.

## Case presentation

A 52-year-old African American male with a history of chronic hepatitis B noncompliant with tenofovir presented with worsening intermittent right upper quadrant abdominal pain for the last two years. Given the increase in the intensity of the pain, he presented for evaluation. The patient denied smoking and illicit drug use and had not consumed alcohol for over 10 years. The progressive nature of the pain prompted further evaluation by his hepatologist. No acute findings were found on examination or laboratory workup. An ultrasound of the liver demonstrated three suspicious lesions. Given these findings, magnetic resonance imaging (MRI) of the abdomen with the liver protocol was obtained, which demonstrated three lesions in the liver; all were LI-RADS 3 (Liver Imaging Reporting and Data System). At baseline, this patient had an ECOG (Eastern Cooperative Oncology Group) performance status of 0. Follow-up MRIs over the next two years portrayed similar-sized lesions and overall stable findings. At the two-year mark, a follow-up MRI demonstrated that the lesion in segment 6 of the right hepatic lobe had increased in size to (1.3x2.2x2 cm) 2.2 cm. This lesion demonstrated arterial enhancement with washout and was categorized as an LI-RADS 5 lesion. He underwent an unsuccessful transjugular liver biopsy. The alpha-fetoprotein (AFP) at the time was 5.7. He was referred to medical oncology at this point. Given the high clinical and radiological suspicion of malignancy, the patient underwent a liver resection to excise the tumor segments. Two hepatic tumors were found and resected, one in segment 6 of the right lobe and another in segment 4b of the left lobe. The lesion in segment 6 was characteristic of both HCC and CC and was thus was termed biphenotypic cHCC-CC. The lesion in segment 4 lobe was moderately differentiated HCC.

The patient recovered well from surgery. Follow-up imaging did not demonstrate evidence of disease. The patient was placed on three to four monthly surveillance visits and scans. Six months later, his lab work demonstrated elevated AFP. MRI of the abdomen showed a liver lesion in the right lobe segment 6/7 measuring 3.6x3.6x3.3 cm with some vascular flow. Computed tomography (CT) of the chest did not demonstrate metastatic disease to the lungs. The patient then underwent a right hepatic lobectomy with a portal lymphadenectomy. The mass resected was HCC, and one out of the three lymph nodes resected was positive for cholangiocarcinoma. He was commenced on chemotherapy with 5-FU and oxaliplatin. After eight cycles of therapy, he developed abdominal pain, which prompted a CT scan of the abdomen. This demonstrated stable disease. Due to the persistent symptoms, a positron emission tomography-computed tomography (PET-CT) was done, which, on the contrary, demonstrated worsening disease. At this point, the option of looking for a clinical trial was explored with the patient. He declined. Given the rising AFP, it was thought that the dominant component is likely the HCC and hence the patient was commenced on nivolumab. He showed some clinical improvement and a stable AFP. After five cycles of therapy, he demonstrated a rising AFP. PET-CT demonstrated significant disease progression. Since he retained an excellent performance status (ECOG 0), we subsequently proceeded with gemcitabine and cisplatin. We attempted to treat both components, HCC and CC, of the disease. Hence, in the setting of disease progression and lack of treatment guidelines, this patient was also commenced concurrently on sorafenib with gemcitabine and cisplatin. Again, he demonstrated clinical stability in the setting of a stable but elevated AFP. He tolerated this therapy very well with a mild hand-foot syndrome, which very quickly responded to salicylic acid 6% cream. A CT scan after three cycles of therapy, along with continued sorafenib treatment, demonstrated stable disease and a slight reduction in the AFP; hence, the current regimen was continued. Unfortunately, at the end of six cycles, imaging demonstrated disease progression and rising AFP. The patient declined a suggestion to explore a clinical trial at a local university hospital.

While he received gemcitabine, cisplatin, and sorafenib, next-generation sequencing (NGS) was performed on his tumor. The results demonstrated a CCND1 genomic alteration, as well as stable microsatellite status and intermediate tumor mutation burden. While there is no Food and Drug Administration (FDA)-approved therapy for a CCND1 genomic amplification in biphenotypic cHCC-CC, Palbociclib is indeed FDA-approved therapy for a CCND1 genomic amplification in hormone-positive, advanced or metastatic breast cancer. After discussing with the patient information available from NGS and lack of data to provide evidence of success with palbociclib in the setting of his disease, the patient agreed to pursue this last option of therapy. He was commenced on palbociclib, which portrayed evidence of decreasing AFPs. The patient continued to maintain a good quality of life for two months that he was on Ibrance. He continued to work full time. Blood counts and other necessary parameters were monitored and remained within acceptable ranges. Unfortunately, two months into being on Ibrance, his disease progressed. During this time, the patient developed bilateral pneumonia and died within a week of his infectious diagnosis from sepsis in the setting of an immunocompromised state.

## Discussion

We present a unique case not only because cHCC-CC is rare cancer but also the clinical avenues this patient’s disease led to are certainly rare and not described in the literature. He had multiple lines of systemic therapy, including chemotherapy, immunotherapy, targeted therapy, and based on the findings of next-generation sequencing, more personalized treatment with a CCND1 inhibitor. His overall survival from the time of is initial surgery to his death was 21 months. Apart from the last couple of weeks, he had a good quality of life and enjoyed an ECOG performance status of 0-1. He was able to continue to work full time during his treatment.

**Figure 1:**
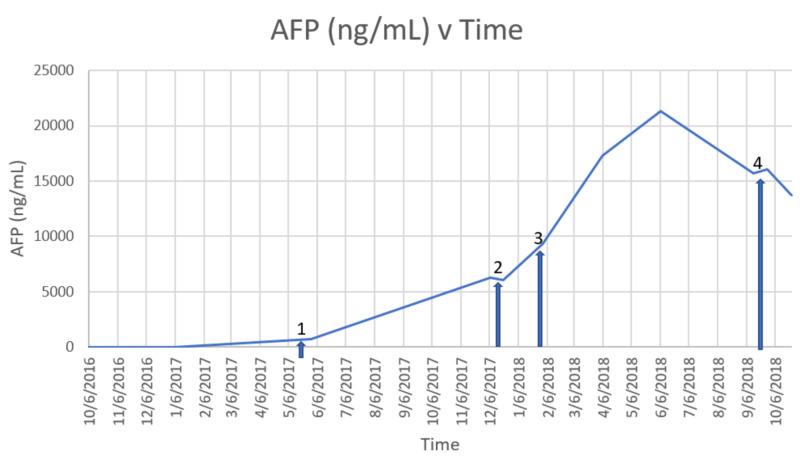
The portrayal of AFP over time with regards to medical intervention 1=initiation of FOLFOX therapy. 2= initiation of Nivolumab. 3=initiation of Cisplatin/Gemcitabine and Sorafenib. 4=initiation of Palbociclib. Most of the medical therapies did not lead to a decrease in AFP. In fact, AFP increased over the course of most of the medical therapy. Palbociclib seems to be the only intervention that leads to a modest decrease in AFP. AFP: alpha-fetoprotein

The rarity of the disease has led to a lack of studies on treatment and treatment-related outcomes. The mean age at diagnosis is 52 years, and there is an 88.9% preponderance for males. Sixty-six point seven percent (66.7%) of the patient's diagnoses with cHCC-CC had previous cirrhosis with 20% of the patients having chronic hepatitis B. The results of a study done by Li and Yang showed that cHCC-CC developed more often in males with already existing chronic hepatitis B-related hepatitis and cirrhosis. The study further showed that the clinical characteristics of cHCC-CC are indeed similar to those of HCC, as in the case with our patient. Our patient displayed arterial enhancement on MRI, which has been portrayed in 27.9%-51.9% of cases on CT. Recent studies also show that 95.6% of cHCC-CC tumors display a washout pattern, as is also the case with our patient. Our patient’s AFP was elevated, which seems to follow the trend that 33%-78% of patients with cHCC-CC also have elevated serum AFP levels, according to this study [2]. Our patient’s rise and falls in AFP are shown below (Figure 1).

Our patient underwent surgery to resect the tumor. Surgical treatment appears to be the mainstay of treatment, as the median survival of patients with cHCC-CC tumors post-resection is 32 months with a five-year survival rate of 24%. The study also reported a median disease-free survival of 16 months in patients with cHCC-CC [3]. According to another study, surgical resection of the mixed cHCC-CC tumor resulted in a three-year survival rate of 34.6%, a 5-year survival rate of 23.1%- and a 10-year survival rate of 11.5% [4-5].

Research regarding systemic therapy in treating cHCC-CC is minimal and appears to only minimally impact survival. One study portrayed that systemic therapy only produced a progression-free survival (PFS) of 3.4 months and overall survival (OS) of 8.3 months. Specifically, sorafenib showed a PFS of 5.5 months and OS of 10.7 months. This study also showed that when treating CC with gemcitabine and cisplatin, patients achieved a PFS of eight months and OS of 11.7 months. However, in the setting of cHCC-CC, the patients portrayed poorer outcomes [5-7]. While cHCC-CC remains a rare neoplastic process, there is much evidence that further research into the diagnosis, management, and treatment paradigms of this pathology is much needed.

## Conclusions

cHCC-CC is a rare neoplastic liver malignancy making up less than 5% of primary liver cancers. Though this is a rare neoplastic process, physicians should be aware of this entity, as there is very little literature to guide patient management. Surgery has proven the most definitive treatment with varied responses to systemic treatment regimens.
